# Towards dielectric relaxation at a single molecule scale

**DOI:** 10.1038/s41598-022-06684-9

**Published:** 2022-02-21

**Authors:** Vitalii Stetsovych, Simon Feigl, Radovan Vranik, Bareld Wit, Eva Rauls, Jindřich Nejedlý, Michal Šámal, Ivo Starý, Stefan Müllegger

**Affiliations:** 1 Institute of Semiconductor and Solid State Physics, Johannes Kepler University Linz, Linz, Austria; 2 Institute for mathematics and physics, University of Stavanger, Stavanger, Norway; 3 Institute of Organic Chemistry and Biochemistry of the Czech Academy of Sciences, Prague, Czech Republic

**Keywords:** Characterization and analytical techniques, Molecular electronics, Surfaces, interfaces and thin films, Molecular electronics, Nanoscience and technology, Nanometrology

## Abstract

Dielectric relaxation lies at the heart of well-established techniques of dielectric spectroscopy essential to diverse fields of research and technology. We report an experimental route for increasing the sensitivity of dielectric spectroscopy ultimately towards the scale of a single molecule. We use the method of radio frequency scanning tunneling microscopy to excite a single molecule junction based on a polar substituted helicene molecule by an electric field oscillating at 2–5 GHz. We detect the dielectric relaxation of the single molecule junction indirectly via its effect of power dissipation, which causes lateral displacement. From our data we determine a corresponding relaxation time of about 300 ps—consistent with literature values of similar helicene derivatives obtained by conventional methods of dielectric spectroscopy.

## Introduction

The phenomenon of dielectric relaxation describes the response of a dielectric substance to an external oscillating electric field and lies at the heart of dielectric spectroscopy^1,2^. The latter is a well-established technique indispensable for diverse fields of research and technology, including molecular sciences, biophysics, medicine as well as petroleum-related industry. Herein we report an experimental route for increasing the sensitivity of dielectric spectroscopy ultimately towards the scale of a single molecule. For this we use the method of radio frequency (RF) scanning tunneling microscopy (STM)^3^. RFSTM combines scanning probe microscopy with complementary concepts borrowed from microwave resonance spectroscopy, resulting in a powerful combination of high spatial resolution of $<10^{-9}$ m with simultaneous energy resolution of $<10^{-7}$ eV for spectroscopy. Recently, RFSTM methods have been developed that utilize an external modulation of the electric field across the tunnel junction at MHz to GHz frequencies; in particular, they have enabled the successful thermometry at the nanometer scale^4^, magnetic resonance spectroscopy at the single spin level^5–7^, noise spectroscopy^8^, addressing single-atom magnets^9,10^, ferromagnetic resonance^11^ as well as spectroscopy of high-frequency mechanical motion of molecules^4,12^.

**Figure 1:**
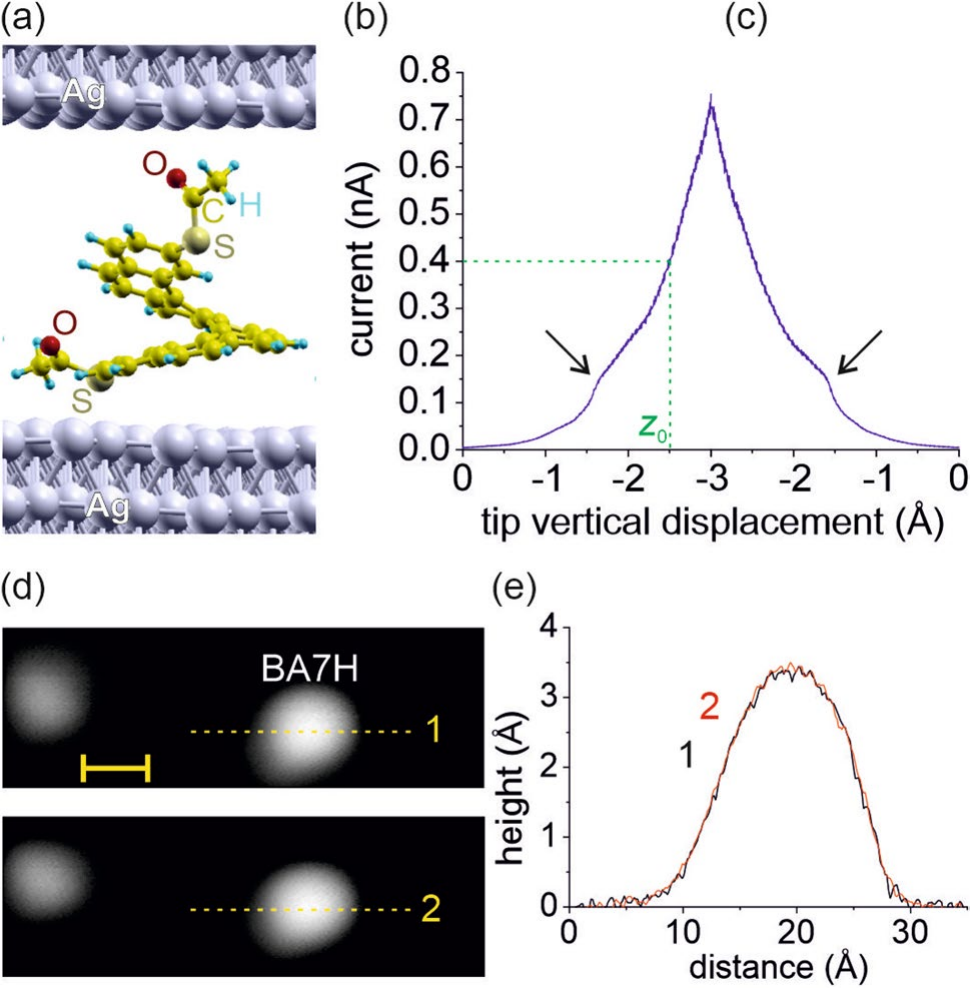
(**a**) DFT simulation of the SMJ formed by a single BA7H molecule covalently bound between two Ag electrodes. (**b**,**c**) Dependence of the current through the SMJ during formation (**b**) and breaking (**c**) of the SMJ by controlled variation of the STM tip-sample separation. (**d**) STM images (scale bar is 1 nm; tunnel conditions: $+0.5$ V, 9 pA; *z* scale is 350 pm) of a single BA7H molecule before formation (top) and after breaking (bottom) of the SMJ. (**e**) Height profiles across the dashed lines labelled 1 and 2 in (**d**) obtained before formation of the SMJ and after breaking the SMJ.

We use RFSTM to excite a polar single molecule junction (SMJ) by an RF electric field of constant amplitude and variable frequency at well-controlled conditions of ultra high vacuum and cryogenic temperatures. As a molecular model system, we investigate the SMJ based on a single molecule of substituted heptahelicene (BA7H) covalently bound between a Ag(111) single crystal surface and the Ag-coated tip of our STM, see Fig. 1a. Similar helicene derivatives were previously studied in SMJs at DC electric fields, in particular for their dependence of conductance and thermopower on mechanical compression^13^ as well as piezoelectric response^14^. With our existing experimental setup we detect the dielectric relaxation indirectly via its effect of power dissipation, which causes lateral displacement of the SMJ. By exploiting our RFSTM as sensitive position detector, we observe a characteristic frequency dependence. Specifically, the SMJ exhibits a mechanical instability (displacement) caused by the RF electric field close to 3.4 GHz. Our results demonstrate that, in principle, RFSTM is capable of performing dielectric spectroscopy at the scale of a single molecule. In particular, our results may impact the fabrication of high frequency single molecule devices in the future^15^.

## Results

### Well-controlled formation and breaking of the SMJ under DC operation

Stetsovych et al.^14^ have studied the SMJ Ag(111)/BA7H/Ag by DC-STM and AFM, revealing that BA7H forms a Ag–S bond with Ag(111) through one acetylsulfanyl group and a Ag–O bond with the STM tip through the other acetylsulfanyl group. Similar to the reported procedure, we have prepared individual BA7H molecules on Ag(111) by separating them from molecular clusters readily found on the sample surface. The separation has been performed with the STM tip using lateral manipulation techniques^16,17^. All our experiments were done on ’bright’ conformers of BA7H^14^, which is clearly identified by its STM topographic height profile. We contact the molecule and form the SMJ by approaching with the Ag-coated STM tip at the position of the upper acetylsulfanyl functional group with a speed of 1.3 Å/s. Positioning of the STM tip over the molecule was performed with a lateral accuracy of $\pm 0.5$ Å. The SMJ is established by the formation of a covalent bond between the upper oxygen atom of the acetylsulfanyl group and a Ag atom of the STM tip^14^. Figure 1a shows the spatial conformation of the SMJ as obtained from our DFT simulations. We control the bond formation by monitoring the electrical tunnel current *I*(*z*) during the contacting by the STM tip. Bond formation typically occurs close to 0.2 nA and is clearly visible as an abrupt increase (kink) in the *I*(*z*) trace, as shown exemplarily in Fig. 1b. Additional *I*(*z*) traces are shown in Fig. S1 of the Supplementary Material. In our experiments we operate the SMJ at a DC electrical resistance of 125 M$\Omega$; we approach the STM tip with a fixed $V_\mathrm {DC}=+50$ mV until *I*(*z*) reaches 0.4 nA at a respective separation $z_0$, see dashed line.

Breaking the SMJ is performed in a well-controlled manner, by retracting the STM tip, which breaks the Ag–O bond between BA7H and the STM tip. Figure 1c shows a typical *I*(*z*) trace during controlled breaking of the SMJ. Careful analysis of our STM images shows that the lateral position of the BA7H molecule is very stable to within $<1$ Å  before and after forming/breaking the SMJ. This value is significantly smaller than the observed RF-field-induced lateral displacement described below.

For every single SMJ reported herein we have carefully verified that it is stable under DC STM operation and robust against $\ge 4$ times forming/breaking operations, in particular, showing negligible lateral displacement. The BA7H molecule undergoes no significant structural change as evidenced by STM imaging, see Fig. 1d,e, and its *I*(*z*) trace during formation/breaking is smooth and free of spikes similar to the curves of Fig. 1b,c.

### Exposing the SMJ to a RF electric field

Previous STM and AFM studies have revealed electromechanic (piezoelectric) response of the SMJ, which was attributed to the interaction of the DC electric field between Ag(111) and the STM tip with a permanent electric dipole moment in the molecule. The latter is oriented perpendicular to the Ag(111) surface as obtained by previous DFT calculations^14^. In this work we investigate the effect of an alternating high-frequency electric field, $E_\mathrm {RF}(t)= (V_\mathrm {RF}/z_0)\cdot \mathrm {cos}(2\pi f\cdot t)$, on the SMJ. $V_\mathrm {RF}$ is the RF voltage amplitude across the SMJ and $z_0$ is the separation between the two Ag electrodes of the SMJ, see Fig. 1.

We apply $E_\mathrm {RF}(t)$ through the tip of our STM instrument, with the frequency *f* ranging from 2 to 5 GHz. We have studied only those SMJs that proved stable under DC-STM operation as described above. In total, we have studied more than 330 individual SMJs. Every SMJ was exposed to an $E_\mathrm {RF}(t)$ pulse as follows: After SMJ formation with $V_\mathrm {DC}=+50$ mV and active STM feedback (constant tunnel current of 0.4 nA), we add a sinusoidal voltage with frequency *f* and amplitude $V_\mathrm {RF}$ for a total pulse duration of 60 s to the STM tip electrode, as described in the Methods section. We set $V_\mathrm {RF} = 2\cdot V_\mathrm {DC}=100$ mV (zero-to-peak) so that the piezoelectric strain of the SMJ can be larger for the RF field than for the DC field. The separation of the SMJ is $z_0\approx 1.4$ nm as predicted by our DFT calculations. Accordingly, $E_\mathrm {RF}(t)$ across the SMJ has an amplitude of $7\times 10^7$ V/m independent of frequency.

After the RF pulse, we break the SMJ (without significantly affecting the molecule, see above) and investigate by STM possible effects of the RF pules on the BA7H molecule. We investigate RF pulsing at 11 different frequency values between 2 and 5 GHz and for each frequency value for $\ge 30$ times with a fresh individual SMJ.

### Effect of the RF pulse

**Figure 2:**
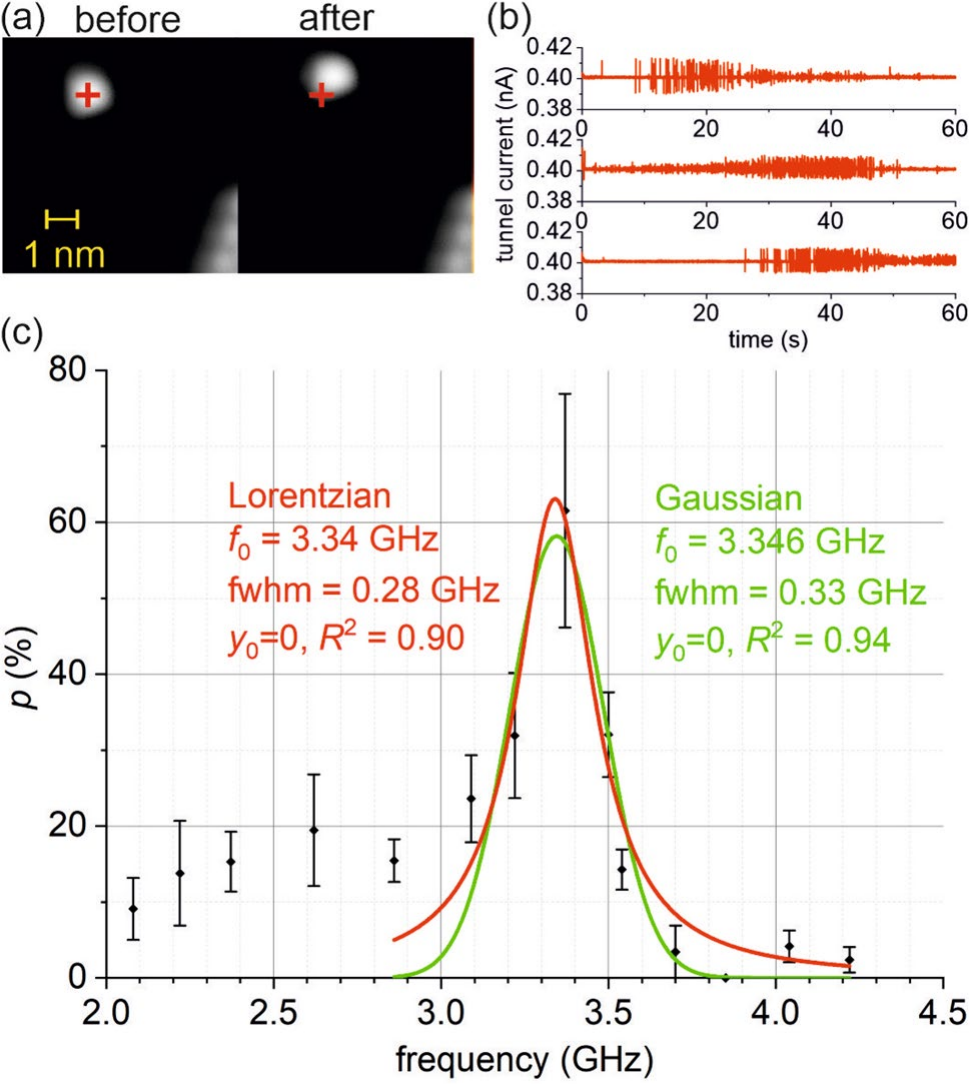
(**a**) STM images of a single BA7H molecule before and after RF pulsing ($7.5 \times 7.5$ nm; z scale 350 pm; tunnel conditions: + 0.5 V 9 pA). The cross marks the initial position of the molecule. (**b**) Typical tunnel current traces during RF pulse for the case that SMJ displacement occurs. (**c**) Frequency dependence of the probability *p* of lateral displacement of the SMJ (see text) for constant RF amplitude of $V_\mathrm {RF}=100$ mV.

In many cases we observe that after applying the RF pulse and breaking the SMJ, the BA7H molecule has been laterally displaced on the Ag(111) surface relative to its initial position by a distance, $\Delta d$, that is significantly larger than any stochastic displacement caused by DC effects as described above. The displacement is readily apparent by STM imaging, as exemplarily shown in Fig. 2a. We found displacements as large as $\Delta d>10$ Å. The observed displacement indicates that the RF pulse causes the BA7H molecule of the SMJ to overcome the surface diffusion energy barriers of both Ag(111) and the STM tip. We remark that the diffusion energy barrier is, in general, considerably smaller than the binding energy of the molecule to the respective electrode of the SMJ, which is here determined by the covalent Ag-S and Ag–O bonds, respectively. Figure 2b shows typical *I*(*t*) traces recorded during RF pulsing, in which lateral displacement has occurred. Clearly, displacement is related to an instability of the SMJ evidenced by current fluctuations. In contrast, current fluctuations were absent in the cases of no displacement.

In our analysis of experimental $\Delta d$ values we define a threshold of $\Delta d \ge 5$ Å  in order to distinguish the displacement caused by RF pulsing from displacement due to other effects. We remark that the threshold value is (1) significantly larger than the upper limit of stochastic DC-induced displacement ($<1$ Å), (2) large enough to allow for unambiguous analysis of our STM images (lateral resolution of $\approx 1$ Å), and (3) significantly ($2\times$) smaller than the typical displacements observed in our RF experiments (up to $>10$ Å), in order to yield expressive statistics.

For each frequency value investigated herein, we have determined from our multiple repeated RF pulse experiments the probability, *p*, that an RF pulse causes lateral displacement by $\Delta d\ge 5$ Å. We calculate the probability as the ratio $p=N_\mathrm {displaced}/N_\mathrm {tot}$, where $N_\mathrm {displaced}$ is the number of experiments where lateral displacement was observed and $N_\mathrm {tot}$ is the total number of experiments.

Plotting *p* against the frequency, we find that *p*(*f*) is frequency-dependent, as shown in Fig. 2c. Clearly, *p* shows a steep increase, reaching a maximum of $>60$% at $f_0=3.37$ GHz, followed by a steep decrease. For $f<<f_0$, *p* has nonzero values between about 10–20%. For $f>>f_0$, *p* is close to zero.

## Discussion

### Dielectric relaxation

Dielectric relaxation originates from a delay in the polarization of the molecule with respect to an alternating-in-time electric field^1,18^. The imaginary part, $\varepsilon ''(f)$, of the dielectric function, $\varepsilon =\varepsilon ' - i\cdot \varepsilon ''$, represents the frequency dependent dissipation of energy. Helicene molecules possess a wide, chiral and polarizable $\pi$ electronic system^19,20^. Our helicene-based SMJ carries a permanent electric dipole moment, $\vec {\mu }_{e,0}$ of about 4.6 Debye at zero electric field, as obtained from our DFT calculations. The permanent dipole moment results from the polar functional groups of BA7H and the electronic interaction with the substrate^14^. In comparison, values between 1 and 7 Debye have also been reported for similar substituted heptahelicene molecules in the gas phase^21,22^. The response of a molecule’s dipole moment to an electric field is determined by its dynamic electric polarizability tensor, $\alpha$, which gives, in linear approximation^23^, the well-known relation $\vec {\mu }_e = \vec {\mu }_{e,0}+ \alpha \cdot \vec {E}$. The period of our exciting RF field, $E_\mathrm {RF}(t)$, defines a characteristic timescale of 1/*f* = 200–500 ps. This is a typical timescale in which the ion density of the molecular backbone—determined by the atomic nuclei and strongly bound electrons—responds to an oscillating electric field. Typical microscopic relaxation times of about 1–330 ps have been reported for small hydrocarbon molecules^24–26^. In comparison, the electrons in the frontier orbitals of the molecule respond almost instantaneously, i.e. within less than a few picoseconds^23,27^.

We adopt the well-known Debye relaxation model^1,18,28,29^ to approximate the response of our SMJ to the time-varying electric field as shown in Fig. 2c. The Debye model predicts a Lorentzian shape of the absorption peak $\varepsilon ''(f) \propto \frac{2\pi f\tau _0}{1+(2\pi f)^2\tau _0^2}$ with relaxation time $\tau _0=\frac{1}{f_0}$ determined by the frequency $f_0$ where absorption reaches its maximum. We have numerically fitted a Lorentzian peak to the experimental *p*(*f*) data, see red curve in Fig. 2c, yielding a good agreement of $R^2=0.90$. From the fit we determine a value of $f_0 = 3.34$ GHz, resulting in a relaxation time of $\tau _0\approx 300$ ps, which seems plausible as compared to literature values (see above).

Numerical fitting with a Gaussian peak (green curve) yields even better agreement of $R^2=0.94$. A Gaussian broadening of the peak may be attributed to the fact that our *p* values are averages, i.e. they do not distinguish how many times (one or more) a displacement of the SMJ has occurred during the RF pulse.

Furthermore, taking into account the finite DC conductivity of the molecule is expected to cause a characteristic asymmetry of the response peak^18^. In particular, the dissipative response at frequencies far below $f_0$ is expected to have nonzero values, while values close to zero are expected at frequencies far above $f_0$. Comparison with the experimental p values of Fig. 2c shows good qualitative agreement. Accordingly, we attribute the nonzero *p* values of about 10–20% observed in our SMJ at frequencies below $f_0$ (see Fig. 2c) to the finite conductance of the BA7H molecule.

**Figure 3:**
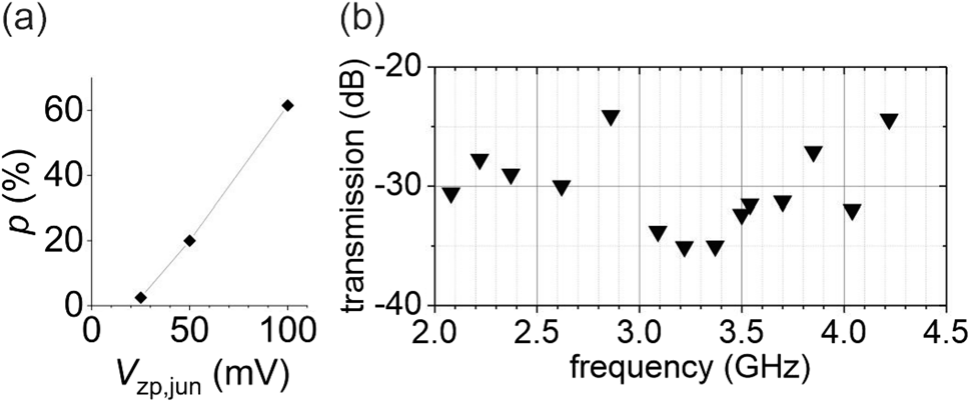
(**a**) Dependence of *p* on $V_\mathrm {RF}$ measured at 3.37 GHz, see text. (**b**) Frequency dependent transmission of our RFSTM instrument in the bandwidth 2–4.5 GHz. Uncertainty is $\pm 1$ dB.

Figure 3a shows the amplitude-dependence of *p* as obtained from experiments at $f=3.37$ GHz. *p* increases slightly over-linearly with increasing $V_\mathrm {RF}$. This result is consistent with dielectric relaxation, considering that *p*, as measured herein, is expected to increase with dissipation, in general. In comparison, Debye relaxation predicts power dissipation to grow with the square of the electric field amplitude^28^.

### Resonance

Figure 2c may be interpreted as mechanical resonance behavior of a damped driven oscillator with resonance frequency close to 3.4 GHz and a quality factor of $Q=f_0/\mathrm {fwhm}\approx 12$. Our DFT calculations yield a lowest frequency of ca. 400 GHz for the mechanical modes of the SMJ and therefore mechanical resonance seems unlikely. To date, only few cases have been reported where indeed single molecule mechanical resonance at GHz frequencies have been observed by STM^12,30,31^. Nevertheless, notice the intriguing coincidence that the rotational constant of an acetylsulfanyl group around the C–S bond axis of BA7H of $\approx 3.5$ GHz lies close to $f_0$.

### Ruling out DC effects and thermal effects

Inelastic excitation by the tunnel electrons under DC electric field may cause stochastic switching of the SMJ, as previously reported by Kitagawa et al.^32^ in a helicene-based SMJ. Stochastic switching is independent of frequency. In contrast, our SMJ clearly exhibits a frequency dependence, thus we rule out stochastic inelastic processes. Notice that our chosen threshold, $\Delta d=5$ Å, for obtaining the *p*-values is significantly larger than the upper limit of DC induced lateral displacement of 1 Å. Thus, we rule out DC-effects such as a static electric field as well as inelastic excitation by tunnelling electrons, in general. We conclude that the observed lateral displacement effect of the SMJ (Fig. 2c) is triggered by the RF pulse, with maximum excitation probability close to 3.4 GHz.

Thermal effects, including the thermally activated diffusion of the BA7H molecule relative to the Ag surface, are expected to show stochastic behavior as well, which is independent of frequency—in clear contrast to the observed frequency dependence. In our experiments we kept the temperature of the STM head constant to $8\pm \,0.5$ K during RF pulsing. As a control experiment, we monitored the lateral position of the SMJ at an elevated temperature of 11 K achieved by ohmic heating the STM for $> 1$ hour, while keeping RF off. We found no lateral displacement greater than the expected tiny DC-induced displacement of max 1 Å. Thus, we rule out thermal effects such as slight increases of $\le 0.5$ K as origin of the observed displacement effect.

Moreover, we found no correlation between the *p*(*f*) curve (Fig. 2c) and the frequency-dependent RF transmission of our RFSTM instrument^33^ shown in Fig. 3b. In particular, we have investigated over pristine Ag(111) possible drift of the *z*-piezo induced by local heating during RF pulsing; this was done at constant-height conditions (feedback off) in order to reveal possible effects of frequency-dependent dissipation in the transmission line. We found that *z*-drift may occur during RF pulsing with $V_\mathrm {RF} = 100$ mV, but we found no correlation with the *p* values of Fig. 2c. Note that RF pulsing of the SMJ was done at constant-current conditions, which compensates for z-drift. Additionally, we observed that RF pulsing has negligible effect on the lateral drift ($\le 2$ Å/h) of our instrument. Thus, we rule out the frequency-dependent damping and related thermal effects as origin of the *p*-plot.

## Conclusion

In summary, the work presented herein is a first proof of principle that radio frequency scanning tunneling microscopy is capable of enabling the experimental measurement of dielectric relaxation at the single molecule level. For our helicene-based single molecule junction we experimentally observe a strongly increased probability of lateral displacement close to 3.4 GHz (Fig. 2c), indicating energy dissipation via dielectric relaxation of the molecular junction. From our data we determine a corresponding relaxation time of about 300 ps. Effects occurring outside or independent of the molecular junction were successfully ruled out. Our method is an important step forward in developing dielectric spectroscopy towards single-molecule sensitivity.

## Materials and methods

Experiments were performed in ultrahigh vacuum at a base pressure of $<10^{-10}$ mbar and a substrate temperature of $\le 8.5$ K. Our instrument is a commercial low-temperature scanning tunneling microscope (CreaTec GmbH, Germany) that we have upgraded with dedicated high-frequency transmission lines for applying AC voltages across the tunnel junction at frequencies between 10 MHz and several GHz^33^. Bias voltage refers to the sample voltage with respect to the STM tip (tungsten, electrochemically etched and thermally deoxidized in ultrahigh vacuum). The STM tip apex was in-situ coated with Ag atoms by controlled indentation into the pristine Ag(111) substrate by $<1$ nm. The state of the STM tip was optimized and carefully checked prior to measurements by reproducing the well-known step-like signature of the Ag(111) surface state^34^. In addition to the commercial DC voltage source of the STM instrument, we couple an independent RF voltage source (Keysight N5173B) to the transmission line via a bias-tee (Tektronix PSPL5541A)^33^. The amplitude of the RF voltage, $V_\mathrm {RF}$, across the SMJ was determined experimentally^33^ similar to the procedure suggested by Krieger et al.^35^ and further developed by Paul et al.^36^. By calibrating the output power of the RF source with frequency we have maintained constant $V_\mathrm {RF}=100$ mV zero-to-peak at the SMJ for variable frequencies throughout the experiments. In addition, to maintain well-controlled contact conditions of the SMJ, a constant DC voltage $V_\mathrm {DC}=+50$ mV was applied.

Racemic *rac*-S,S‘-heptahelicene-2,17-diyl diethanethioate BA7H^14^ was synthesized from *rac*-2,17-dichloroheptahelicene^14^ by a modified procedure: A Schlenk flask flushed with argon was charged with sodium methanethiolate freshly prepared from sodium (257 mg, 11.2 mmol, 50 equiv.) and dimethyl disulfide (653 mg, 0.620 mL, 6.93 mmol, 31 equiv.) in freshly distilled *N*-methyl-2-pyrrolidone (5 mL). A degassed solution of rac-2,17-dichloroheptahelicene (100 mg, 0.224 mmol) in freshly distilled *N*-methyl-2-pyrrolidone (3 mL) was added and the reaction mixture was stirred at 200 $^{\circ }$C for 4 h. After cooling to room temperature, acetyl chloride (1.32 g, 1.2 mL, 16.8 mmol, 75 equiv.) was added and the mixture was stirred at room temperature for 2 h. It was poured into ice, extracted with dichloromethane ($3\times 20$ mL), and the organic layers were washed with brine ($3\times 20$ mL). The solvents were evaporated under reduced pressure and the residue was purified by flash chromatography (cyclohexane-dichloromethane 40:60–0:100) to afford rac-BA7H (76 mg, 65%) as a yellow solid. The $^{1}\mathrm {H}$- and $^{13}\mathrm {C}$- NMR spectra were in agreement with the literature^14^.

Samples were prepared under ultrahigh vacuum conditions (pressure $\le 6\times 10^{-10}$ mbar) at 300 K and in-situ transferred to the STM. The substrate is a commercial Ag(111) single crystal (SPL Inc.). Pristine Ag(111) surface has been prepared by standard procedures of repeated cycles of $\hbox {Ar}^+$ ion sputtering (600 eV) followed by thermal annealing at 720 K. BA7H molecules were deposited by thermal evaporation from a quartz crucible onto the Ag(111) surface held at a temperature of 413 K. The nominal coverage was about 0.1 monolayers.

Density functional theory (DFT) calculations were performed using the Vienna Ab initio Simulation Package (VASP)^37^ for structural relaxation. We employed the gradient-corrected PW91 functional^38^ to account for the exchange and correlation interactions. Dispersion interactions have been taken into account by using the DFT-D scheme^39^. The electron-ion interaction was described by pseudopotentials within the projector-augmented wave (PAW) method^40^, which allows for a moderate energy cutoff of 400 eV for the plane-wave basis. The adsorbate consisting of a BA7H molecule adsorbed in different configurations on the Ag surface and the Ag-tip has been calculated using periodic boundary conditions. The complete ad-system was modeled by periodically repeated supercells, containing slabs of six Ag layers separated by a vacuum of a thickness of 22–24 Å. The topmost two layers and all atoms of the molecules were allowed to relax freely, whereas the atoms in the two center layers were kept fixed at the ideal bulk positions. Due to periodic boundary conditions along the surface normal, the two bottom layers of the Ag-slab serve as STM-tip and were also allowed to relax freely. The structural relaxations were performed with convergence criteria of 0.03 eV/Å  and $10^{-5}$ eV for forces and total energies, respectively. Due to the large lateral supercell sizes, $16.7 \times 14.7$ Å  (one molecule per unit cell), the Brillouin zone could be sampled with the Gamma point.

## Supplementary Information

Supplementary Information.
