# Supplementary material for: Towards dielectric relaxation at a single molecule scale
Source: Sci Rep. 2022 Feb 21;12:2865. doi: 10.1038/s41598-022-06684-9 (PMC8861178; doi:10.1038/s41598-022-06684-9)
Supplement: Supplementary file 1 — Supplementary Information. [file 41598_2022_6684_MOESM1_ESM.pdf]

Supplementary Material:

Towards dielectric relaxation at a single molecule scale

Vitalii Stetsovych[1], Simon Feigl[1], Radovan Vranik[1], Bareld Wit[1], Eva Rauls, Jindrich Nejedly, Michal Samal, Ivo Stary, and Stefan Müllegger[1]

[1] Institute of Semiconductor and Solid State Physics, Johannes Kepler University Linz, Austria.

[2] Institute for mathematics and physics, University of Stavanger, Norway.

[3] Institute of Organic Chemistry and Biochemistry of the Czech Academy of Sciences, Prague, Czech Republic.

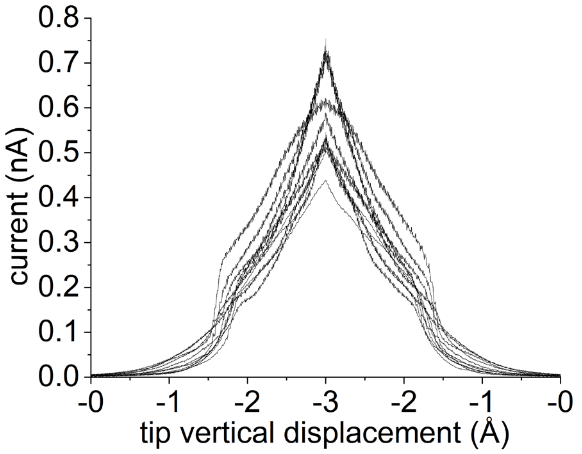

Figure S1. Formation/breaking curves of single molecule junction (SMJ) recorded on 11 different bright monomers of BA7H/Ag(111) (see main text). Dependence of the current through the SMJ during formation (left) and breaking (right) of the SMJ by controlled variation of the STM tip-sample separation.
